# Identifying interventions to improve hand hygiene compliance in the intensive care unit through co-design with stakeholders

**DOI:** 10.12688/hrbopenres.13296.2

**Published:** 2021-07-16

**Authors:** Kathryn Lambe, Sinéad Lydon, Jenny McSharry, Molly Byrne, Janet Squires, Michael Power, Christine Domegan, Paul O'Connor

**Affiliations:** 1 Health Research Board, Grattan House, 67-72 Lower Mount Street, Dublin 2, D02 H638, Ireland; 2 School of Medicine, National University of Ireland Galway, Co. Galway, H91 TK33, Ireland; 3 Irish Centre for Applied Patient Safety and Simulation, School of Medicine, National University of Ireland Galway, Co. Galway, H91 TK33, Ireland; 4 Health Behaviour Change Research Group, School of Psychology, National University of Ireland Galway, Co. Galway, H91 TK33, Ireland; 5 The University of Ottawa, Ottawa, ON, K1N 6N5, Canada; 6 The Ottawa Hospital Research Institute, Ottawa, ON, K1H 8L6, Canada; 7 National Clinical Programme for Critical Care, Clinical Strategy & Programmes Division, Health Service Executive, Dublin, D02 X236, Ireland; 8 J.E. Cairnes School of Business and Economics, National University of Ireland Galway, Co. Galway, H91 TK33, Ireland; 9 Discipline of General Practice, National University of Ireland Galway, Co. Galway, H91 TK33, Ireland

**Keywords:** Critical care, intensive care, infection control, hand hygiene, hand disinfection, co-design

## Abstract

**Background:** Despite the effectiveness of hand hygiene (HH) for infection control, there is a lack of robust scientific data to guide how HH can be improved in intensive care units (ICUs).  The aim of this study is to use the literature, researcher, and stakeholder opinion to explicate potential interventions for improving HH compliance in the ICU, and provide an indication of the suitability of these interventions.

**Methods: **A four-phase co-design study was designed. First, data from a previously completed systematic literature review was used in order to identify unique components of existing interventions to improve HH in ICUs. Second, a workshop was held with a panel of 10 experts to identify additional intervention components. Third, the 91 intervention components resulting from the literature review and workshop were synthesised into a final list of 21 hand hygiene interventions. Finally, the affordability, practicability, effectiveness, acceptability, side-effects/safety, and equity of each intervention was rated by 39 stakeholders (health services researchers, ICU staff, and the public).

**Results:** Ensuring the availability of essential supplies for HH compliance was the intervention that received most approval from stakeholders. Interventions involving role models and peer-to-peer accountability and support were also well regarded by stakeholders. Education/training interventions were commonplace and popular. Punitive interventions were poorly regarded.

**Conclusions:** Hospitals and regulators must make decisions regarding how to improve HH compliance in the absence of scientific consensus on effective methods. Using collective input and a co-design approach, the guidance developed herein may usefully support implementation of HH interventions that are considered to be effective and acceptable by stakeholders.

## Introduction

Healthcare-associated infections (HAIs) present a serious challenge to safe, effective, and efficient healthcare. HAIs are of particular concern in the Intensive Care Unit (ICU), where prevalence rates of between 20% and 30% have been reported ^1^. Research suggests that more than half of HAIs may be preventable ^2^, with appropriate hand hygiene (HH) considered to be the most effective safeguard ^3^. Despite the importance of hand hygiene (HH), there are a number of weaknesses ^4, 5^ in the research evidence to guide the implementation of HH interventions:

- Lack of methodological rigour. There is a lack of methodologically robust studies to explore the effectiveness of interventions to increase HH compliance ^4– 6^;
- Lack of a theoretical basis for intervention. Safety interventions commonly fail to have a theoretical basis to support the implementation of evidence into practice ^7, 8^ and HH interventions are no exception ^6^;
- Lack of practical guidance in how to apply and sustain good HH practices. There are few descriptions of concrete and practical strategies to translate these guidelines to everyday practice in ICUs ^8^, and;
- Inadequate understanding of the complexities of the environment and organisation in which the behaviour is to take place. Changes in an organisation require the consideration of a range of factors interacting at different levels of an organisation ^7^.

These weaknesses mean that decisions regarding HH improvement are made in the absence of scientific consensus ^4^. The purpose of this study is to take a co-design approach in order to leverage the knowledge of subject area experts and other relevant stakeholders to provide guidance on pragmatic interventions for improving HH compliance in ICU settings. Co-design in healthcare involves the equal partnership of those who work within the system (healthcare staff), those who use the system (patients and their families/carers), and those who design interventions for the system (e.g. healthcare staff, quality improvement specialist, researchers), with a shared goal of achieving better outcomes or more efficient processes ^9^.

The Behaviour Change Wheel ^10, 11^ was used to provide a theoretically grounded and structured approach to intervention development. The Behaviour Change Wheel is a framework for understanding behaviour and developing interventions to target behaviour change. This framework has been used in previous studies of HH ^12– 14^. This framework specifies nine intervention functions, which describe ways that behaviour may be targeted and changed ^10^. These functions were used in the current study to support the development and analysis of HH interventions identified in the co-design activities. The intervention functions ^10^ are:

- Coercion: creating an expectation of punishment or cost;
- Education: increasing knowledge or understanding;
- Training: imparting skills;
- Enablement: increasing means or reducing barriers to increase capability, or opportunity;
- Environmental restructuring: changing the physical or social context;
- Incentivisation: creating an expectation of reward;
- Modelling: providing an example for people to aspire to or imitate;
- Persuasion: using communication to induce positive or negative feelings or stimulate action; and
- Restriction: using rules to reduce the opportunity to engage in the target behaviour.

An intervention- such as those that promote peer-to-peer accountability and support among staff in the ICU- has the function of enablement (increasing means or reducing barriers to increase capability or opportunity). It is also important to point out that each intervention has a number of ‘intervention components’. These components are the discrete elements that make up an intervention. Using the peer-to-peer accountability and support example, these components may include encouraging staff to remind one another about HH and the appointing of role models. The specific components of each intervention may be implemented in whatever combination is most applicable to a particular ICU.

The overall aim of the study was to derive a list of possible interventions to enable infection control teams to identify an intervention that will address hand hygiene compliance in their own ICU.

The specific objectives of the study were to:

- derive a comprehensive list of HH intervention components from the existing literature and input from an international panel of researchers;
- synthesise the intervention components into a list of unique interventions, each with a central focus and possible variations; and
- to elicit feedback on the suitability of each of these interventions from a panel of stakeholders, including members of the public, ICU staff, and researchers.

This co-design study was carried out in four stages: 1) a systematic review; 2) an expert panel workshop; 3) synthesis of intervention components; and 4) a stakeholder survey. The methods and results from each of these stages is described below, followed by a general discussion.

## Stage 1: data extraction from systematic literature review

The purpose of the systematic literature review was to synthesize the literature describing interventions to improve HH in ICUs, to evaluate the quality of the extant research, and to outline the type, and efficacy, of interventions described. This systematic review has been published previously ^15^. Therefore, only summary information on this systematic review are provided below.

### Systematic literature review: methods

We used data from our previously completed literature review in order to identify existing interventions to improve HH in ICUs ^15^. Electronic searches of five databases were carried out in order to identify peer-reviewed studies evaluating any interventions to improve hand hygiene in adult ICU settings. Intervention components for all included interventions were extracted by three of the review authors using the intervention functions of the Behaviour Change Wheel ^10^. Study quality was assessed using the Downs and Black Checklist ^16^.

### Systematic literature review: results

The results have been described in more detail elsewhere ^15^. In summary, 38 studies were identified (see *Underlying data* ^17^), employing 76 different HH intervention components. The intervention components most commonly fell under the intervention functions of education (79%), enablement (71%), training (68%), environmental restructuring (66%), and persuasion (66%). Studies were generally found to have poor methodological rigour, and frequently evaluated the effects of several components at the same time (e.g., education plus rewards). Intervention outcomes were variable, with a mean relative percentage change of 95%. However, the use of bundled interventions makes it difficult to determine the effect of individual components. Best practice for improving compliance, therefore, remains unestablished.

## Stage 2: expert panel workshop

The purposes of the expert panel workshop were to: 1) identify additional intervention components not identified in the systematic literature review carried out in Stage 1; and 2) to elaborate on the details of implementation, strengths, challenges, and potential targets across a range of HH interventions. This stage was underpinned by a content analysis approach

### Expert panel workshop: methods

***Recruitment and participants.*** A purposive sampling strategy was used to recruit participants for the expert panel workshop. Efforts were made to include representative participants from a diversity of academic backgrounds, healthcare professions, and countries with a broad range of expertise, as well as patient representatives. Potential participants were approached via email and invited to take part. A total of 10 of the 11 invitees agreed to attend and four of those were from outside Ireland. The one person who declined had another engagement on the day of the workshop. Five had a background in the social sciences, four in nursing, and one was a patient advocate. All of the nurses had clinical experience, with one with specific ICU experience. Eight of the participants had previously published research in the area of behaviour change and/or infection control.

***Ethics statement.*** Ethical approval was received from National University of Ireland, Galway’s Research Ethics Committee (ref: 19-Mar-27) and written informed consent to participate in the workshop was obtained from all participants (via signature).

***Procedure.*** The one-day workshop took place in June 2019 at the National University of Ireland, Galway. Three of the authors (POC, a lecturer, a man, >15 years health services research; SL, a lecturer, a woman, >10 years health services research; and KL, a post-doctoral researcher, a woman, >5 years health services research) served as facilitators to guide the discussion and record the proceedings. The participants in the workshops were known to the facilitators, and no-one else was present at the workshops besides the participants and researchers. There were three parts to the workshop.

1. Introduction (1 hour). The workshop opened with a round of introductions. This was followed by a presentation by the workshop facilitators, outlining the purpose of the project, work achieved to date, including the systematic review data, and an outline of the intervention design task- to include providing an overview of the intervention functions from the Behaviour Change Wheel. Consistent with co-design principles ^9^, the contributions of the facilitators were short and flexibility was built into the workshop so that the expert panel could set priorities and direct the agenda.
2. Small group intervention development exercise (2 hours). The workshop participants were divided into small groups, each with a workshop facilitator. The goal of these groups was to generate ideas for interventions. Each group was assigned two or three intervention functions from the Behaviour Change Wheel and asked to generate ideas for intervention components under each of these functions based on their professional experience, knowledge of the research literature, and/or clinical experience. To aid in the intervention generation process, the groups were provided with worksheets with the following prompts: describe the intervention component idea; describe the target group; identify the strengths and challenges of the intervention; identify any cost implications; and identify appropriate outcome measures.
3. Discussion and refinement (2 hours). Workshop participants reconvened to share their ideas and comment on the intervention components in a round-table discussion format. The discussion sessions were audio-recorded, and handwritten notes were taken by the facilitators. The worksheets completed by the small groups were also retained for analysis. The audio recordings were not transcribed. The purpose of the recordings was as a back-up to the written notes should anything be unclear or require clarification.

### Expert panel workshop: results

The data collected from the workshops was entered, collated, and organised using Microsoft Excel 2016. The data collected from the workshop resulted in 16 intervention components. The audio recordings of the workshops were not used as the facilitator notes as the worksheets were sufficiently detailed (see *Underlying data* ^17^). The expert panel emphasised the social environment as a key point of leverage for improving HH compliance (n=7 interventions), particularly advocating a collaborative approach among staff and infection control teams to goal setting and action planning (n=4). Suggestions were also made around improving team working in specific difficult clinical situations, assigning role models, and “charming nagging” to encourage compliance among peers. The importance of good role models and well-chosen HH “champions” to shape the approach to HH on the unit was also emphasised; these individuals should be well-respected, approachable, and encouraging. The interventions were largely generated under the Environmental Restructuring function (n=5). Other interventions included providing feedback on compliance and carefully using emotional or fear-based messaging to emphasise the consequences of poor compliance.

## Stage 3: synthesis of intervention components

The purpose of this third stage was to collate the intervention components derived from the first two stages of the study and to synthesise them into a detailed list of interventions, which could be feasibly presented for evaluation to the stakeholder survey group in the final stage of the study.

### Synthesis of intervention components: methods

The intervention components derived from the literature review (102 components) and the expert panel workshop (16 components) were collated, categorised using the intervention functions of the Behaviour Change Wheel, and synthesised into 91 unique intervention components (see *Underlying data*, ^17^). An iterative process was used to group similar components. For example, the components ‘public display of compliance rates’, ‘unit-level feedback’, and ‘monthly report cards emailed to chiefs of service’ were collapsed into a single intervention entitled ‘monitoring and feedback at unit level’. The synthesis process was carried out with the following goals in mind:

- it was determined by the authors that the number of interventions should not be so large as to be unwieldy or unusable in the context of a toolkit to improve HH compliance; and
- the specificity and detail of the individual components should be preserved insofar as is practical, to ensure that the final interventions remain useful and actionable.

For each intervention, a summary description was prepared of the core intervention concept, possible variations where appropriate, strengths and challenges, and cost implications. This process was carried out using Microsoft Excel 2016 by two members of the researcher team (POC and KL), and then independently reviewed by another member of the team (SL). These summaries were composed based on learning from the research literature and expert panel discussion, the experience of the research team, and the unique components that were collapsed into each intervention.

### Synthesis of intervention components: results

The iterative synthesis process outlined above collapsed the 91 unique intervention components into a final list of 21 interventions (see Table 1 and *Underlying data* for a more detailed description of each intervention).

**Table 1.** Final list of interventions and intervention functions (as categorised using the Behaviour Change Wheel ^10^).

Intervention	Intervention function	Source
Proactive corrective action *Corrective action for unsatisfactory compliance; additional education,* * clarification, reinforcement, disciplinary action, standardised process*	Coercion	Review
Warning letters *Warning letters issued for staff members who are repeatedly noted to be* * negligent in hand hygiene compliance*	Coercion	Review
Comprehensive active education and training for hand hygiene *High standard of comprehensive education and training in basic skills and* * knowledge, regular top-up sessions*	Education/training	Review & workshop
Simulation training for hand hygiene *Simulation and debriefing in a supportive learning environment using* * mannequins, artificial models, UV light equipment, etc.*	Education/training	Review
Tailored education and training for professional groups *Segregated training groups, taking account of challenges and role* * conceptualisations unique to each professional group*	Education/training	Workshop
Ongoing / top-up education and training *Refresher education and training actively provided on a continuous basis*	Education/training	Review
Continuous education through visual communications *Use of printed materials and multimedia to reinforce the hand hygiene* * message, e.g., strips, mirrors, posters, promotional materials, etc.*	Education/training	Review & workshop
Peer-to-peer accountability and support *Encouragement and normalisation of friendly reminders, feedback and* * support for good practice between peers*	Enablement	Review & workshop
Get staff feedback on the alcohol hand gel to be made available in units for hand cleansing *Different types of gel trialled on ward and staff feedback solicited to inform* * procurement decisions and foster sense of ownership*	Enablement	Review
Ensure availability of essential supplies for hand hygiene behaviour *Adequate availability of necessary supplies for hand hygiene is ensured,* * monitored and maintained closely, and assessed in audits*	Enablement	Review
Demonstrated support for hand hygiene from hospital leadership *Hospital directors, leaders and senior management show support for hand* * hygiene efforts and emphasise its importance*	Environmental restructuring	Review & workshop
Hand hygiene breaks *Regular breaks scheduled during which all staff on the ward pause their* * work, where it is safe to do so, and thoroughly wash their hands*	Environmental restructuring	Review
Support for improving the local institutional safety culture *Positive institutional safety culture is fostered on a broader level, improving* * norms, values, and basic assumptions about safety*	Environmental restructuring	Review
Consultation with frontline staff about hand hygiene improvement *Action plans are developed based on staff feedback on barriers to hand* * hygiene compliance, appropriate and realistic targets, etc.*	Incentivisation	Review & workshop
Competitions, prizes and rewards *Material rewards for satisfactory compliance and achieving targets; coffee,* * lunch, recognition ceremonies, friendly competitions between wards, etc.*	Incentivisation	Review & workshop
Providing strong hand hygiene role models within professional groups *Leaders recruited to provide a good example to staff, model good hand* * hygiene behaviour, and offer reminders, recognition, and informal praise*	Modelling	Review & workshop
Screening and identification of patients carrying MRSA and other “superbugs” *Patients screened on admission to identify carriers of “superbugs” so that* * staff may implement additional precautions as necessary*	Persuasion	Review
Monitoring and feedback at unit level *Hand hygiene compliance monitored at unit level with feedback to staff,* * public display of performance metrics, inter-unit comparisons, etc.*	Persuasion	Review
Monitoring and feedback for individual staff members *Hand hygiene compliance monitored at individual level with specific* * feedback and advice for improvement*	Persuasion	Review & workshop
Inclusion of hand hygiene behaviour in all procedural protocols *Guidelines for hand hygiene included in any relevant protocols, including* * equipment, techniques, ward setup, mandatory training, etc.*	Restrictions	Review
Implementation of universal contact precautions during outbreaks of serious infectious illness *Additional precautions (use of single rooms, additional cleaning and PPE* * requirements) for all patients during outbreaks of serious infectious illness*	Restrictions	Review

## Stage 4: stakeholder survey

The purpose of the stakeholder survey was to systematically elicit input from a range of stakeholders on the utility of the 21 HH intervention described in Table 1 using the ‘APEASE’ criteria (affordability, practicability, effectiveness, acceptability, side-effects/safety, and equity). This is a set of criteria set out in the Behaviour Change Wheel guidance to assist intervention developers in evaluating behaviour change interventions ^10^.

### Stakeholder survey: methods

***Recruitment and participants.*** A purposive sampling strategy was used to recruit stakeholders by directly emailing potential participants to ask them to complete the survey. Everyone who was contacted agreed to participate. Representatives were recruited from three specific groups: public representatives, ICU staff, and health services researchers. Members of the public and ICU staff received a €100 voucher for their participation. The final sample (n=39) included 11 members of the public, 11 ICU doctors (mean 6.7 years of experience), 10 ICU nurses (mean 16.4 years of experience), and seven health services researchers (mean 13.2 years of experience). The number of respondents was pragmatic, based upon the number of participants we were able to recruit in the time available, and for which there was funding for incentives. Of the respondents to the survey, two health service researchers and one member of the public also participated in the expert panel workshop.

***Ethics statement.*** Ethical approval was received from the National University of Ireland, Galway’s Research Ethics Committee (ref: 18-Sept-17) and written informed consent was obtained from all participants to participate and use their data in the study via a tick box.

***Procedure.*** The survey was carried out using SurveyMonkey, and online survey software (Google Forms is a freely available alternative). The participants were provided with brief background information on the study in a five-minute video introduction. The 21 interventions outlined in Table 1 were then presented in a random order to each participant. For each intervention, the participants were provided with the intervention summary description (see *Underlying data* ^17^). The survey was distributed during May 2020.

For each intervention, the participants were asked to rate their agreement with each of the following five APEASE dimensions on a 0 (strongly disagree) to 100 (strongly agree) slider scale:

- Affordability: intervention can be delivered within an acceptable budget.
- Practicability: intervention can be delivered with minimal disruption to patient care.
- Effectiveness: intervention is likely to improve hand hygiene compliance.
- Acceptability: intervention will be considered appropriate by staff in the ICU.
- Side effects/safety: intervention will not have any unwanted side-effects or unintended consequences.
- Equity: intervention can be delivered in any ICU in the Republic of Ireland.

A free-text box was also provided for each intervention for any additional comments. Statistical analysis was completed using IBM SPSS version 21. For each intervention, the mean of the responses to each of the five APEASE dimensions was calculated. An overall mean APEASE score for each intervention was then calculated by deriving the mean of the dimension scores. A total of 2.4% (n=119 responses) of the data was missing. No imputation methods were used to account for the missing data. One-way ANOVA was conducted to examine differences in scores for each intervention across the three participant groups. For each of the intervention functions, a mean APEASE score was also calculated (e.g., for enablement, the mean of the scores for the three enablement interventions was calculated).

### Stakeholder survey: results

The mean APEASE scores for the 21 interventions ranged from 53.5-81.3 on a scale of 1-100, and are shown in descending order in Table 2, with the individual APEASE scores for each intervention provided in *Underlying data* ^17^. The highest-scoring interventions were ‘ensuring availability of essential supplies’, ‘providing strong hand hygiene role models within professional groups’, and ‘comprehensive active education and training’.

**Table 2.** Final list of interventions with functions and mean APEASE (affordability, practicability, effectiveness, acceptability, side-effects/safety, and equity) scores (range 0–100).

Rank	Intervention	Intervention function	Mean APEASE score (SD)
1	Ensure availability of essential supplies for hand hygiene behaviour	Enablement	82.0 (14.6)
2	Providing strong hand hygiene role models within professional groups	Modelling	74.0 (16.1)
3	Comprehensive active education and training for hand hygiene	Education/ training	73.5 (15.5)
4	Continuous education through visual communications	Education/ training	73.4 (18.0)
5	Peer-to-peer accountability and support	Enablement	72.5 (16.2)
6	Monitoring and feedback at unit level	Persuasion	71.2 (17.3)
7	Ongoing / top-up education and training	Education/ training	70.5 (16.9)
8	Tailored education and training for professional groups	Education/ training	70.4 (18.4)
9	Support for improving the local institutional safety culture	Environmental restructuring	70.3 (18.6)
10	Inclusion of hand hygiene behaviour in all procedural protocols	Restrictions	70.3 (17.7)
11	Implementation of universal contact precautions during outbreaks of serious infectious illness	Restrictions	69.9 (21.1)
12	Screening and identification of patients carrying MRSA and other “superbugs”	Persuasion	69.3 (22.21)
13	Consultation with frontline staff about hand hygiene improvement	Incentivisation	68.9 (18.3)
14	Simulation training for hand hygiene	Education/ training	67.6 (16.7)
15	Proactive corrective action	Coercion	66.7 (18.3)
16	Competitions, prizes and rewards	Incentivisation	66.2 (17.0)
17	Get staff feedback on the alcohol hand gel to be made available in units for hand cleansing	Enablement	65.4 (22.4)
18	Demonstrated support for hand hygiene from hospital leadership	Environmental restructuring	64.9 (1.4)
19	Monitoring and feedback for individual staff members	Persuasion	60.2 (19.2)
20	Warning letters	Coercion	59.1 (18.8)
21	Hand hygiene breaks	Environmental restructuring	53.9 (18.4)

The intervention function with the highest mean APEASE score was Modelling (74.0), although only one of the included interventions fell under this intervention function. The next highest were Enablement (73.6, three interventions) and Education/training (71.0, five interventions). Environmental restructuring (62.9, three interventions) and Coercion (73.2, two interventions) received the lowest mean ratings.

No significant differences were found in mean APEASE scores between the three groups of participants. The free-text responses to the interventions were mixed. Concerns that were most frequently raised included that the interventions may create a negative atmosphere of scrutiny or be difficult to implement in a fair, transparent way. A summary of the free-text responses about each intervention is provided in *Underlying data* ^17^.

## Discussion

While regulators prioritise improvement in HH and specify ‘what’ standards must be achieved, there is also a need for practical guidance on ‘how’ these standards can be achieved ^18^. The relative lack of guidance on how to improve HH compliance is exacerbated by the paucity of strong evidence to identify what interventions are likely to be effective ^15^, and how potential interventions should be implemented to meet the needs of specific ICUs. However, despite the lack of robust research evidence, there is still a need to provide advice and guidance to hospital managers and clinicians on how best to invest limited resources to support improvements in HH compliance. With that advice in mind, the purpose of this study was to use the available literature, expert and stakeholder opinion to identify potential interventions for improving HH compliance in the ICU, and to give some indication of the suitability and acceptability of these interventions.

Ensuring the availability of essential supplies for HH behaviour was the intervention that received most approval from stakeholders. Interventions involving role models and peer-to-peer accountability and support were also well regarded. Education/training interventions were commonplace and popular. Punitive interventions were poorly regarded. It is perhaps unsurprising that the need to ensure the availability of essential supplies for HH behaviour was the highest-scoring intervention in the stakeholder survey. The World Health Organization recommends the use of alcohol-based hand rub at the point of care ^19^. The availability of essential supplies is the most fundamental HH intervention, and any other interventions are likely to have limited effectiveness if essential supplies are unavailable. Not withstanding the problems with limited personnel protective equipment at the beginning of the COVID-19 pandemic, the lack of supplies is less of an issue for high-income countries like, Ireland. However, the same may not be true in lower income countries. A recent systematic review of levels of HH compliance in ICU settings found that healthcare practitioners were 65% compliant in high-income countries, as compared to 9% compliant in low-income countries- at least partially due to poor resourcing ^20^. The limitations of the products used for HH, such as alcohol-based handrub (e.g. drying time, odour, skin irritation, ease of use) have also been identified as barriers to HH compliance ^21^, and this is suggested as an area of future research and development. It is further suggested that regular audits are conducted in ICU units to ensure that HH supplies are always available for ICU staff, visiting healthcare teams, and members of the public/patients.

The provision of strong HH role models within professional groups was the second highest-scoring intervention and is related to peer-to-peer accountability and support (ranked fifth). Ownership and leadership were the most notable enablers of HH compliance mentioned by Irish HH policy makers in a recent study ^8^. Healthcare workers have reported that they frequently adjust their behaviour to match those that they see in clinical practice, and can feel strongly influenced to abstain from compliance by negative role models ^22^. This suggests that positive role models can also have the same effect. This positive motivation could be achieved by encouraging senior staff members, particularly consultants and senior nursing staff, to act as role models for junior staff members ^23^. However, it is important that in such interventions the role of different levels of leadership (e.g., senior clinical staff, management) and styles of leadership are clear if the intervention is to be effective ^24^. Given the positive disposition of the stakeholders to these types of intervention, as well as the relative lack of research on this approach ^15^, it is suggested that the efficacy of this approach should be examined in future research.

Five of the 21 HH interventions identified in this research were concerned with the education and training of healthcare practitioners. These interventions received favourable ratings from the stakeholders. Given that 79% of studies in our systematic literature review were concerned with education, and 69% with training interventions, it is clear that these approaches are ubiquitous in HH improvement programmes ^15^. However, ICU staff frequently report having the requisite knowledge and skills to carry out HH, which may mean they are resistant to mandatory HH training ^12, 25^. Therefore, it is suggested that there is a need for targeted HH training in which ICU staff receive individualised and direct feedback on their HH performance at the bedside. Individual feedback is supported by a number of studies ^26, 27^. Therefore, it is recommended that, at least for experienced ICU staff, that there is a shift from an approach of standard training delivered to all staff, to one that is tailored to the needs of specific units. To illustrate, in a recent study carried out across three Irish ICUs, it was found that nurses were more likely to engage in HH than other healthcare professionals, and the ICU staff observed were 2.6 times more likely to comply with HH if the indication for HH was self-protective rather than patient-protective ^28^. We have developed a toolkit to support ICU staff to tailor HH interventions to the specific needs of the unit. The toolkit provides guidance to ICU staff on how to choose a suitable intervention for improving HH compliance in their unit, implement the intervention, and assess whether the intervention has been effective29. However, future research should examine the effectiveness of the toolkit to tailor HH training and education for specific units and groups.

Two of the interventions that were identified and reviewed in this study involved coercion or reprimands- proactive corrective action and warning letters. Both of these potential interventions were unfavourably rated by the stakeholders, and these punitive approaches are likely to be ineffective. In fact, they may lead to a tendency for individuals to then cover up any errors that are made and are counter to the ‘just culture’ most healthcare organisations wish to foster. A just culture approach recognises that healthcare professionals make errors and may take shortcuts or fail to follow protocols ^30^. There may be good reasons why staff do not follow procedures (e.g., not following HH protocols during an arrest due to the urgency of delivering care). In a just culture, it is recognised that there is a need to understand why healthcare professionals make errors and the importance of encouraging honest reporting from healthcare workers as to why things may go wrong. Identifying issues with the system is an important precursor in order to develop a tailored HH intervention; future research into co-design and collaborative approaches to doing so would be extremely advantageous.

### Strengths and limitations

The strengths of this study are that it utilised a multiple-methods approach with input from the research literature, experienced researchers, ICU staff and members of the public. However, there are a number of limitations of this research that should be acknowledged. First, there were no health service managers or regulators in either the expert panel or as survey participants. Therefore, the perspective of people in these roles are not represented. Second, the stakeholder survey participants were all based in Ireland, which may lead to questions about the generalisability of our findings to other countries. However, as Ireland’s HH compliance figures are broadly in line with those in other high-income countries ^28^, we believe that the findings can be generalised to ICU settings in other high-income countries. Also, four of the members of the expert panel were from outside of Ireland. Third, the recommendations and assessments of the HH interventions are based upon opinion rather than upon scientific evidence for their effectiveness. This is certainly true, and can be attributed to the absence of robust HH research that can be considered sufficiently rigorous to support the identification of effective interventions ^15^. However, in the absence of sufficiently rigorous studies, our findings provide some direction on the HH interventions that should be considered based upon the collective opinions of the international experts and stakeholders that participated in this study. Finally, and related to the previous limitation, we did not evaluate the effectiveness of any of the HH interventions. This was beyond the scope of co-design study. However, future research is needed to focus on assessment of intervention effectiveness.

## Conclusions

In conclusion, despite the large financial and logistical investments required to implement a HH intervention, hospitals and regulators must make decisions regarding infection control policies in the absence of scientific consensus on what is effective. However, by using the collective input from a range of stakeholders, it is hoped that some guidance can be provided as to the HH interventions that are at likely to be suitable and acceptable (or not) to stakeholders, and encourage the rigorous evaluation of these HH interventions in future studies.

## Data availability

### Underlying data

Zenodo: Identifying interventions to improve hand hygiene compliance in the ICU through co-design with stakeholders. http://doi.org/10.5281/zenodo.4778492 ^17^.

This project contains the following underlying data:

- Upload to open access v3.xlsx (references of the papers described in the systematic review (stage 1), the collated notes from the expert workshop (stage 2), synthesis (stage 3) and the anonymised questionnaire data (stage 4)).
- Intervention and ratings.pdf (description of the 21 interventions, APEASE ratings, and stakeholder qualitative comments).

Data are available under the terms of the Creative Commons Attribution 4.0 International license (CC-BY 4.0).
