# Supplementary material for: Identifying interventions to improve hand hygiene compliance in the intensive care unit through co-design with stakeholders
Source: HRB Open Res. 2021 Jul 16;4:64. Originally published 2021 Jun 10. [Version 2] doi: 10.12688/hrbopenres.13296.2 (PMC8243226; doi:10.12688/hrbopenres.13296.2)
Supplement: Peer review history

# Peer review: Reviewer response for version 1

**DOI:** 10.21956/hrbopenres.14472.r29656

**Published:** 2021-06-29

**Authors:** Marie Ward, David Vaughan

**Affiliations:** 1 Centre for Innovative Human Systems, School of Psychology, Trinity College Dublin, Dublin, Ireland; 2 St. James's Hospital, Leeds, UK; 3 Children's Health Ireland (CHI), Dublin, Ireland

This is a very interesting study which addresses an area of critical importance in healthcare - hand hygiene and infection prevention control. A co-design approach is taken with mixed methods employed to identify which components of the Behaviour Wheel Change might be most effective in improving hand hygiene compliance in the ICU. It describes an approach that allows interested parties to understand the gap between "work as imagined vs work as done", and by describing the barriers in a systematic way, to attempt to overcome these barriers.

 The study is well thought out and builds on previous work carrying out a systematic review of the area. The working of the expert panel is well described and in keeping with co-design; the stakeholders for the survey are a good mix of public, ICU staff and researchers. A minor point, but perhaps to be considered in future such work - there might be a benefit in the inclusion of the following in co-design workshops: representatives of regulators and manager; the former because it is easier for regulators to mandate changes without understanding the barriers to achieving them, and the latter because theoretically, they control the resource to enable delivery.

 It is an interesting finding that the simplest things matter a lot in terms of influencing behaviour - actual supply and availability of PPE. It would be interesting to see reference to the PPE situation at the start of the COVID-19 pandemic in the discussion but we appreciate that this paper precedes that timeframe. 

**Some minor comments include: **

- Introduction - definition of co-design - researchers are just one example of those who design interventions to improve the healthcare system.
- Methods - Stage 2 - Expert panel participants - clarify if any of the participants have clinical experience or experience of working in an ICU setting.
- Stage 3 - synthesis of intervention components - 22 components are mentioned from the expert panel workshop. Earlier in Stage 2 - expert panel workshop results 16 components are mentioned.
- Discussion - strengths and limitations - under generalisability it is worth noting that 4 of the expert panel participants did come from outside Ireland.

Is the work clearly and accurately presented and does it cite the current literature?

Yes

If applicable, is the statistical analysis and its interpretation appropriate?

Yes

Are all the source data underlying the results available to ensure full reproducibility?

Yes

Is the study design appropriate and is the work technically sound?

Yes

Are the conclusions drawn adequately supported by the results?

Yes

Are sufficient details of methods and analysis provided to allow replication by others?

Yes

Reviewer Expertise:

Human Factors; Patient Safety

We confirm that we have read this submission and believe that we have an appropriate level of expertise to confirm that it is of an acceptable scientific standard.

# Response

**Published:** 2021-07-13

**Authors:** Paul Paul

**Affiliations:** National University of Ireland, Galway, Ireland

This is a very interesting study which addresses an area of critical importance in healthcare - hand hygiene and infection prevention control. A co-design approach is taken with mixed methods employed to identify which components of the Behaviour Wheel Change might be most effective in improving hand hygiene compliance in the ICU. It describes an approach that allows interested parties to understand the gap between "work as imagined vs work as done", and by describing the barriers in a systematic way, to attempt to overcome these barriers.

**Thank you.**

 The study is well thought out and builds on previous work carrying out a systematic review of the area. The working of the expert panel is well described and in keeping with co-design; the stakeholders for the survey are a good mix of public, ICU staff and researchers. A minor point, but perhaps to be considered in future such work - there might be a benefit in the inclusion of the following in co-design workshops: representatives of regulators and manager; the former because it is easier for regulators to mandate changes without understanding the barriers to achieving them, and the latter because theoretically, they control the resource to enable delivery.

**This is a good point, and would agree that there is great benefit to including regulators and managers in a co-design group. This has been acknowledged as a limitation in the revised manuscript.**

 It is an interesting finding that the simplest things matter a lot in terms of influencing behaviour - actual supply and availability of PPE. It would be interesting to see reference to the PPE situation at the start of the COVID-19 pandemic in the discussion but we appreciate that this paper precedes that timeframe. 

**We thought about including this. Apart from the stakeholder survey, all of the work was carried out pre-pandemic. Therefore, even the content of the stakeholder survey was based on pre-pandemic research. However, we have now added in the discussion that lack of appropriate PPE was an issue in Ireland at the beginning of the COVID-19 pandemic.**

 Some minor comments include: 

 Introduction - definition of co-design - researchers are just one example of those who design interventions to improve the healthcare system.

**This is certainly true. In the revised manuscript we have changed this to “e.g. healthcare staff, quality improvement specialists, researchers”.**

 Methods - Stage 2 - Expert panel participants - clarify if any of the participants have clinical experience or experience of working in an ICU setting.

**All five of the nurses had clinical experience, and one was an ICU nurse. This point has been clarified in the revision.**

 Stage 3 - synthesis of intervention components - 22 components are mentioned from the expert panel workshop. Earlier in Stage 2 - expert panel workshop results 16 components are mentioned.

**This inconsistency in numbers is a typo – apologies and thank you for spotting this. The correct number is 16. We have corrected this error.**

 Discussion - strengths and limitations - under generalisability it is worth noting that 4 of the expert panel participants did come from outside Ireland.

**We have added this point as recommended.**

# Peer review: Reviewer response for version 1

**DOI:** 10.21956/hrbopenres.14472.r29653

**Published:** 2021-06-25

**Authors:** Judith Dyson

**Affiliations:** 1 School of Health Sciences, Birmingham City University, Birmingham, UK

Thank you for approaching me to review this study. I very much enjoyed reading this, I will certainly use your findings in my own work and imagine many others working in the fields of implementation science, improvement, clinical practice (whether IPC or ICU) will too. 

 Your review identified existing interventions to improve HH in in ICU and you did this using the intervention functions of the BCW. I wondered. How did you find this approach? Was it difficult? Any particular problems with this approach. Did they all fit the framework?

 Your approach is interesting and valid, I wondered why you chose to categorise to intervention functions rather than behaviour change techniques (an approach I have seen others take)?

 Your co-design workshops looked really thorough. Did the patient representative have any unique views or ideas? With the intervention development exercise – I see you didn’t share the did you intervention components designed/published in the literature.

 In table 1 I see you have one element that wasn’t identified in the literature – tailoring of training according to group, which I thought was both novel and insightful. I also liked the way you applied the APEASE criteria. 

 When did you conduct your groups? (Sorry if I missed this – couldn’t see it). Was it during COVID-19 or pre? I wondered because the top intervention was availability of supplies. I thought we had nailed that challenge in most of the Western world – I appreciate you refer to other countries too. On that note – you talk about the need for tailoring; how might that be done with your intervention components?

 What are you as a team going to do with the results of this study? It would be great to see you test these interventions in practice. 

 A final note - love the term “charming nagging”. Whoever came up with that is a genius!

Is the work clearly and accurately presented and does it cite the current literature?

Yes

If applicable, is the statistical analysis and its interpretation appropriate?

Not applicable

Are all the source data underlying the results available to ensure full reproducibility?

Yes

Is the study design appropriate and is the work technically sound?

Yes

Are the conclusions drawn adequately supported by the results?

Yes

Are sufficient details of methods and analysis provided to allow replication by others?

Yes

Reviewer Expertise:

Implementation Science, Behaviour change, Improvement Science, Infection Prevention

I confirm that I have read this submission and believe that I have an appropriate level of expertise to confirm that it is of an acceptable scientific standard.

# Response

**Published:** 2021-07-13

**Authors:** Paul Paul

**Affiliations:** National University of Ireland, Galway, Ireland

Thank you for approaching me to review this study. I very much enjoyed reading this, I will certainly use your findings in my own work and imagine many others working in the fields of implementation science, improvement, clinical practice (whether IPC or ICU) will too. 

**We are delighted to hear that you will use the findings, and that you also think others will as well.**

 Your review identified existing interventions to improve HH in in ICU and you did this using the intervention functions of the BCW. I wondered. How did you find this approach? Was it difficult? Any particular problems with this approach. Did they all fit the framework?

**The interventions all fit within the framework as it is very comprehensive. Some thought was sometimes required to decide the most appropriate intervention function. We also combined education and training into one function as it was not generally possible to tease these two functions apart.**

 Your approach is interesting and valid, I wondered why you chose to categorise to intervention functions rather than behaviour change techniques (an approach I have seen others take)?

**The focus of the study, and in fact, the research project as a whole was on the identification of higher level intervention functions, rather than the more granular levels of behaviour change techniques. Therefore, the focus was more upon identifying specific interventions. We felt that focusing on intervention functions, rather than behavioural change techniques, would be more salient for ICU personnel. We added a line to make this point to the third paragraph of the introduction.**

 Your co-design workshops looked really thorough. Did the patient representative have any unique views or ideas? With the intervention development exercise – I see you didn’t share the did you intervention components designed/published in the literature.

**The patient representative did not share any unique ideas. However, we were pleased that they were active participants in the discussion. We did in fact provide a short overview of the intervention components during the workshop introduction. We have made this explicit in the revised manuscript.**

 In table 1 I see you have one element that wasn’t identified in the literature – tailoring of training according to group, which I thought was both novel and insightful. I also liked the way you applied the APEASE criteria.

**Thank you.**

 When did you conduct your groups? (Sorry if I missed this – couldn’t see it). Was it during COVID-19 or pre? I wondered because the top intervention was availability of supplies. I thought we had nailed that challenge in most of the Western world – I appreciate you refer to other countries too. On that note – you talk about the need for tailoring; how might that be done with your intervention components?

**The co-design workshop was conducted prior to the COVID pandemic (June 2019), and the stakeholder survey was conducted during the pandemic (May 2020). As we describe in the discussion, we believe that the availability of supplies has largely been addressed in developed countries. However, it may still be relevant in other countries. More work is required on how to tailor interventions to the needs of specific ICUs - we have suggested this as an area of future research. However, we have added a reference and short discussion in paragraph 4 of the discussion of a toolkit we have developed which provides guidance on: identifying a suitable intervention for improving HH compliance in an ICU; implementing the intervention; and assessing whether the intervention has been effective. We believe this toolkit will support ICUs in tailoring interventions to their needs and agree with the reviewer that this is key.**

 What are you as a team going to do with the results of this study? It would be great to see you test these interventions in practice. 

**We agree - this is something that we hope to do in the future once ICUs have hopefully returned a bit more to normality. We have added this point to the fourth paragraph of the discussion.**

 A final note - love the term “charming nagging”. Whoever came up with that is a genius!

**We also like this term. It came from one of the nurses in the co-design workshop.**
